# Resilience: supporting children’s self-regulation in infant and toddler classrooms

**DOI:** 10.3389/fpsyg.2024.1271840

**Published:** 2024-02-05

**Authors:** Diane M. Horm, Shinyoung Jeon, Denise Vega Ruvalcaba, Sherri Castle

**Affiliations:** ^1^Early Childhood Education Institute, University of Oklahoma-Tulsa, Tulsa, OK, United States; ^2^Department of Human Development and Family Science, College of Education & Human Development, University of Missouri, Columbia, MO, United States; ^3^Child Trends, Bethesda, MD, United States

**Keywords:** resilience, self-regulation, infant-toddler classrooms, QCIT, teacher-child interactions

## Abstract

**Introduction:**

Resilience is a process that develops as a complex transaction as children experience and shape their social-ecological contexts. The dynamic development of self-regulation is an aspect of resilience that has received increased attention as a key mechanism predicting a variety of important short- and long-term outcomes. The current study examined how the self-regulation skills of infants and toddlers in a classroom could potentially shape classroom interactions and quality which, in turn, could potentially shape the development of self-regulation skills of the individual infants and toddlers enrolled in the classroom across an early childhood program year. The unique contribution of this study is the focus on a critical component of resilience, self-regulation, in an understudied age group, infants and toddlers, in an important and understudied context, the infant-toddler early childhood classroom.

**Methods:**

Data are from a statewide evaluation of early childhood programs serving children birth to age 3 growing up in low-income contexts. Multi-level mediation models were employed to examine the mediation effect of classroom quality between classroom-level self-regulation and individual children’s gain in self-regulation over a year.

**Results:**

We found a significant indirect path. The results showed that classroom-level self-regulation skills demonstrated by infants and toddlers in the fall predicted higher levels of teachers’ implementation of three important aspects of classroom quality – support for social-emotional, cognitive, and language development – in the winter. We also found that higher levels of teachers’ support for social-emotional, cognitive, and language development associated with children’s increased growth in self-regulation skills from fall to spring. The direct path from classroom-level self-regulation demonstrated in the fall to individual children’s gain in self-regulation was not significant.

**Discussion:**

These findings, unique due to the focus on infants and toddlers in a classroom context, are discussed within the larger body of existing self-regulation research conducted with older children and prevalent theories outlining developmental mechanisms. Implications for both infant-toddler classroom practices and future research are addressed. Relative to practice, our findings have implications for informing how the development of self-regulation, an important component of resilience, can be supported in the youngest children, infants and toddlers, specifically those enrolled in center-based classrooms serving young children growing up in families with low incomes. We focus on the need to improve the support and professional development of infant-toddler teachers which, in turn, can improve classroom quality and foster resilience in infants and toddlers. Relative to research, our use of a relatively new measure of infant-toddler classroom quality, the Quality of Care for Infants and Toddlers (QCIT), shows how this tool can expand infant-toddler research, a need in the current literature. Future research using different measures, designs, analytical strategies, and diverse samples and contexts is needed to further explain very young children’s development of self-regulation, a critical component of resilience.

## Introduction

Resilience has been defined in a variety of ways (Masten and Cicchetti, 2016) but commonly is conceptualized as the capacity to successfully adapt to challenges and risks that threaten the individual (Masten and Barnes, 2018). For the developing child, resilience reflects the ability to positively adapt to demands and contexts through various processes and connections (Masten and Barnes, 2018). Resilience involves factors within and external to the child. Included on the “shortlist” of research-based factors promoting resilience in young children are: sensitive caregiving, emotional security, self-regulation skills, self-efficacy, routines, engagement in a well-functioning school, among others (Masten and Barnes, 2018). Although the literature base is growing, there currently remain many open questions about the specific processes and transactions among these factors that result in resilience in different developmental contexts. The purpose of this study is to investigate the dynamic development of self-regulation, an aspect of resilience that has received increased attention as a key mechanism predicting a variety of important outcomes (McClelland et al., 2015), in an understudied age group, infants and toddlers, in an important and understudied context, the early childhood classroom.

## Self-regulation

Self-regulation has also been defined in a variety of ways and calls have been issued to integrate the many definitions that emphasize various components including emotional regulation, self-control, executive function, and effortful control under a broader self-regulation umbrella term (Jones et al., 2016; Nigg, 2017). This broad approach to defining self-regulation encompasses the multiple distinct and overlapping mechanisms that lead to a child’s skill to manage both their behavior and emotions in adaptive ways (Morawska et al., 2019). Blair and Diamond’s (2008) definition, an example of this broad approach, posits self-regulation as a process through which individuals regulate their emotions and behaviors to positively adjust in social relationships and various environments to produce positive outcomes (Blair and Diamond, 2008).

### The importance of self-regulation

Consistent with this definition, research has documented short- and longer-term outcomes associated with self-regulation as a resilience factor in early childhood. For example, Hardaway et al. (2012) found that family chaos was negatively associated with children’s self-regulation which in turn influenced externalizing behaviors. Their findings indicated that children’s self-regulation played a full mediating role in the relationship between family chaos and children’s externalizing behavior. This suggests that children’s self-regulation skills could serve as a crucial factor in mitigating externalizing behaviors within chaotic family environments. Sciaraffa et al. (2018) conceptualized that children’s self-regulation served as a protective factor promoting resiliency in learning, behaviors, and health associated with toxic stress caused by adverse childhood experiences. In fact, MacPhee et al. (2015) have reviewed multiple studies showing self-regulation acts as a resilience factor impacting children’s developmental outcomes. They reviewed multiple intervention programs designed to promote emotional regulation which was related to children’s behavior problems, adolescents’ substance use, and parents’ depression and anxiety. These studies highlight that self-regulation skills can function as a resilience factor under conditions of family adversity including families’ economic hardship.

Prior research has also documented the importance of self-regulation in the school context. For example, McClelland et al. (2006) found that children who enter kindergarten with high self-regulation skills also show higher achievement in math and literacy through sixth grade, with the academic achievement gaps increasing through second grade between children with higher and lower levels of kindergarten self-regulation. Using a large, nationally representative Australian sample, Howard and Williams (2018) found that self-regulatory skills demonstrated in preschool and primary grades were significantly associated with a range of adolescents’ outcomes in academic achievement (reading, numeracy), school truancy, health (weight, smoking, alcohol use), and mental well-being a decade later. This study also documented the potential for changes in self-regulation relatively early in life, between ages 4 and 7, to support positive developmental outcomes by reducing the risk of poor academic achievement and demonstration of risk behaviors that begin in adolescence (e.g., suicidal thoughts, smoking, property crimes, and school truancy) and have significant consequences for lifelong health and well-being.

As illustrated by these examples, a body of research has established that early self-regulation predicts important short- and long-term outcomes across a wide range of areas including school readiness, academic achievement, feelings of higher self-worth, a better ability to cope with stress, better mental health, and more positive adolescent and adult outcomes (Moffitt et al., 2011; McClelland et al., 2015; Montroy et al., 2016; Howard and Williams, 2018). This body of research has prompted some to conclude that the development of self-regulation in young children is an early life marker for later life success (Montroy et al., 2016). Importantly, this body of research also documents that self-regulation is malleable, sensitive to developmental processes to enhance resiliency. Several theories have postulated about the underlying developmental processes.

### The development of self-regulation: theory and past research

Developmental Systems Theory (DST) proposes that individual development emerges from myriad interactions across system levels, all shaped by the interaction of processes within and between individuals and their contexts, and at multiple levels of systems including culture, society, and ecology (Masten and Barnes, 2018). Through this dynamic process, resilience and its development then become not only a feature of the individual child, but rather the complex experiences the child has through relationships and interactions with other people in their system. Accordingly, self-regulation—and resilience, more broadly—within a child shapes the environment and interactions they experience, which in turn, shapes the ongoing development of self-regulation and overall resilience. For example, Blair (2009) emphasizes that age-related changes in self-regulation and individual differences in self-regulation at a given age or stage fundamentally shape children’s experiences and the responses that children evoke from caregivers and others, which further shape self-regulation.

To date, most research on the development of self-regulation has been conducted with preschool age children versus younger children. Previous research suggests a qualitative shift at age 3 with rapid, nonlinear development due to the integration of individually developing components of self-regulation (Diamond, 2002; Montroy et al., 2016). This observation and other research findings document that self-regulation is developing during the infant-toddler period and is impacted by the child’s context.

### The development of self-regulation: the infant-toddler period

The existing literature focused on infants and toddlers has typically examined the home/family context and its associations with the development of early self-regulation. For example, Morawska et al. (2019) note that young infants have very limited capacity to self-regulate but rapidly develop it in response to parental socialization. Between 12 and 18 months of age children develop self-regulation skills (Kochanska et al., 2000) with evidence that specific parent–child interactions foster its development (Valcan et al., 2017). Specifically, meta-analyses document that parental use of positive strategies are associated with better child self-regulation with use of negative parenting strategies (e.g., coercive behaviors) being associated with weaker child self-regulation (Valcan et al., 2017). Even those sources highlighting parental impacts acknowledge the role of more distal contexts including features of the home, non-parental care settings, and peer groups (McCabe et al., 2004).

Despite this acknowledgement, few studies have examined the role of infant-toddler group care in the development of self-regulation. One of the few exceptions (Salminen et al., 2021) examined associations between teacher–child interaction quality and children’s self-regulation in Finnish and Portuguese toddler classrooms and found that specific features of teachers’ classroom interactions can promote the development of children’s self-regulation skills in these two sociocultural contexts. Given the increasing enrollment of infants and toddlers in out-of-home center-based early care and education (ECE) programs in the US (Horm et al., 2016), with infants spending significant time in these settings (Forry et al., 2018), a gap exists in our understanding of the development of self-regulation in this important infant-toddler context.

## High-quality early care and education

While there is scant research about the development of self-regulation in infants and toddlers enrolled in center-based ECE, a large body of research has examined the impact of center-based ECE programs on short- and longer-term outcomes of young children. Overall, high-quality ECE has been found to impact the development of young children significantly and positively, across a variety of outcomes, with effects being larger for those young children growing up in low-income contexts or experiencing other circumstances associated with developmental risks (Barnett and Frede, 2010; McCoy et al., 2017; Meloy et al., 2019). Throughout this body of research, features associated with enhanced child outcomes include aspects of program quality and age of entry and duration of enrollment in high-quality ECE.

Past research documents that classroom quality for infants and toddlers includes both structural and process features (Norris and Horm, 2015, 2016), with the general conclusion that the structural aspects (e.g., ratio, group size) provide the foundation for the expression of process quality embodied by positive interactions, nurturing relationships, and meaningful activities. In a group setting, relationships (Horm et al., 2016) are viewed as key and a large body of evidence documents the importance of high-quality adult-child interactions to later development (e.g., Burchinal et al., 1996; Bratsch-Hines et al., 2020). Past research supports associations between teachers’ sensitive-responsive interactions and numerous positive outcomes for infants and toddlers including increases in positive social skills, receptive and expressive language, and school readiness concepts (see Norris and Horm, 2015 for a summary).

High-quality ECE classrooms support these features through intentional caregiver practices and curriculum. Recommended classroom practices (NAEYC, 2020) and common measures of classroom quality designed for infant and toddler settings both emphasize sensitive, responsive teacher-child interactions as the hallmark of high-quality infant-toddler care and education. As outlined by NAEYC (2020), this means that early childhood educators understand and incorporate three core considerations to inform their classroom practices: understanding of typical, normative child development, understanding of each individual child’s unique characteristics and experiences, and understanding of the broader contexts impacting development and learning.

Peers also play a role in infant-toddler development (Hay et al., 2009) and past research suggests they likely influence classroom practices associated with the development of self-regulation in center-based ECE classrooms. For example, in a study of preschool age children, Choi et al. (2018) found that preschoolers in classrooms with higher average self-regulation skills demonstrated larger gains in teacher-reported self-regulation skills at the end of the school year. Montroy et al. (2016) reported similar findings with another sample of preschoolers. These authors speculated that peers’ self-regulation skills likely affected other children in the same classroom by influencing the teachers’ choices of classroom activities, available materials, classroom routines, and instructional practices. They argued that if the average level of self-regulation in classrooms tends to be high that teachers implementing developmentally appropriate practices (NAEYC, 2020) would be more likely to appropriately adjust classroom activities and expectations that require higher self-regulation skills for children, which, in turn, provides more opportunities for children to practice and further develop these skills.

Past research suggests that early participation in high-quality ECE, starting in infancy, can foster positive development (Zaslow et al., 2010). For example, Li et al. (2013) found that children growing up in low-income contexts had the highest levels of school readiness if they attended high-quality ECE during both their infant-toddler and preschool years. Similarly, Yazejian et al. (2015) found both age of entry and duration of enrollment were positively associated with receptive language at age 5, with stronger effect sizes for dual language learners. These findings imply that children’s experiences in infant-toddler center-based ECE classrooms have large and lasting effects, especially for children considered educationally at-risk. In fact, Campbell et al. (2001) have noted that ECE programs starting in infancy have demonstrated some of the largest and longest lasting impacts. This conclusion, along with the acknowledgement that infancy is a period of rapid growth and change, and thus offers the greatest opportunities for early experiences or interventions to impact the course of development (Raikes et al., 2004), provides a motivation for studying the development of self-regulation in infant-toddler classrooms.

Other motivations include the observation by Howard and Williams (2018) that the conditions to optimally improve self-regulation remain unknown. These authors also note the research base supports the early malleability of self-regulation and potential efficacy of early intervention approaches to strengthen self-regulation. With increasing numbers of infants and toddlers attending ECE programs (Horm et al., 2016), more research is called for to examine how self-regulation skills potentially affect teacher-child interaction, a central component of classroom quality, which in turn, is associated with the positive development of infants and toddlers, theoretically including their self-regulation skills. Substantial research has shown that positive classroom quality is associated with preschool-aged children’s self-regulation skills (Mashburn et al., 2008; Broekhuizen et al., 2016), and studies are needed to explore the impact of classroom quality on the development of self-regulation skills in infants and toddlers.

## This study

The current study addresses open questions regarding underlying processes supporting the development of self-regulation and identified gaps in the current literature with little research focused on the infant-toddler age group, especially in ECE classrooms despite their growing participation in such settings. Another unique contribution is the use of a relatively new observation tool, the Quality of Care for Infants and Toddlers (QCIT; Atkins-Burnett et al., 2015), specifically developed to measure the quality of caregiver-child interactions in ECE group settings serving infants and toddlers. The purpose of this study was to examine how the self-regulation skills of infants and toddlers associate with classroom quality as measured by teacher-child interactions which, in turn, potentially shapes the development of self-regulation skills in infants and toddlers across an ECE program year. Specifically, this study investigated two research questions:

1. Does the classroom-level average of self-regulation skills demonstrated by infants and toddlers associate with gains in individual infant-toddler’s self-regulation across a year of enrollment in an infant-toddler classroom?
2. If the classroom-level average of children’s self-regulation is associated with classroom quality, does this, in turn, associate with further gains in individual infant-toddler’s self-regulation across a year of enrollment in an infant-toddler classroom?

Based on theory and past research, especially that conducted to investigate self-regulation development of preschool-age children in ECE classrooms, we predict the average entry self-regulation skills demonstrated by infants and toddlers will be directly associated with gains in individual infant-toddler’s self-regulation (RQ1). Second, we predict that classroom quality will mediate the link between classroom-level average of self-regulation skills and the ongoing development of self-regulation skills in infants and toddlers (RQ2).

## Materials and methods

### Data and sample

The data used in this study were drawn from a larger evaluation of a statewide initiative supporting early childhood programs serving children birth to age 3 growing up in low-income contexts in the state of Oklahoma (OECP, 2021–2022). The participating programs met the Early Head Start Performance Standards, operated at least 8 hours per day for at least 44 weeks of the year, and staffed each classroom with two teachers with at least one of these teachers holding a minimum of a CDA credential. A total of 253 classrooms across 9 agencies participated in this statewide initiative during 2021–22. From these participating classrooms, the program evaluator selected a total of 52 classrooms to be part of their 2021–22 evaluation study including infant, toddler, and preschool classrooms. Because the research questions posed in the current study focus on the potential associations of infant-toddler classroom quality and the development of self-regulation as a component of resilience in young children from low-income families, we selected only the 37 infant and toddler classrooms that were part of the larger evaluation study. The 37 infant and toddler classrooms represented three large Early Head Start/Head Start agencies in two large metropolitan cities in Oklahoma. Children who were enrolled in infant-toddler classrooms over a year were included in the final analyses (*n* = 269).

### Participants

As noted above, the 269 children included in this study were enrolled in infant or toddler classrooms participating in a statewide initiative (OECP, 2021–2022) to expand and enhance ECE programming for children ages birth to 3 growing up in low-income contexts. As shown on Table 1, the participants included boys (55%) and girls (45%) between the ages of 3 to 37 months. Our sample included Spanish-speaking children (33%) and was racially and ethnically diverse.

**Table 1:** Descriptive statistics.

	Total (Child *n* = 269; Class *n* = 37)		
	M (Range)	SD	Factor loading
Child outcomes (level-1)			
Self-regulation (Fall)	99.53 (67–133)	19.14	
Self-regulation (Spring)	102.47 (67–133)	17.74	
Child characteristics (level-1)			
Gender (1 = male)	55%		
Home language (1 = Spanish)	33% Spanish		
	67% English		
Race/ethnicity	48% Hispanic		
	32% Black		
	20% White		
Classroom characteristics (level-2)			
Infant and toddler classroom quality			
Support for social-emotional development	5.01 (3.13–6.63)	0.86	0.91
Support for cognitive development	3.90 (2.43–5.00)	0.71	0.64
Support for learning development	4.66 (3.30–6.40)	0.77	0.72
Classroom averaged self-regulation skills fall	102.59 (76–133)	12.86	

Due to the fact that this study was a secondary analysis of program evaluation data, additional demographic information on the specific participants was not available. However, the initiative required that participating families met the eligibility criterion of having incomes at or below 185% of the federal poverty level. The statewide initiative reported that for the 2021–2022 school year, 3,011 children were enrolled in rural and urban settings across the state and that a total of 12,023 referrals for basic needs, emergency interventions, and other community service assistance were made (OECP, 2021–2022). This state program was modeled on Early Head Start in terms of eligibility and services provided and thus the families and children enrolled have the demographic characteristics and needs of a population that is marginalized and low resource.

### Design

The design of this study involved accessing existing data on children’s social-emotional development over a program year and information about the quality of the infant-toddler classrooms participating in the state initiative. Standardized measures were used to collect these existing data. Teachers rated children’s social-emotional development, including self-regulation, at the beginning and end of the 2021–22 year. Classrooms were observed in the winter to document quality.

### Procedures

Children’s self-regulation was rated twice, fall and spring, by their infant-toddler classroom teacher. These ratings were completed electronically by each classroom’s lead teacher. Teachers experienced a brief training on how to use the rating scale prior to completing their ratings. Teachers were given 1 month to complete the ratings for the children in their classrooms. Each teacher rated approximately 3 to 17 infants and toddlers, depending if the child’s age conformed to the age range measured by the social-emotional rating scale used in the larger evaluation study. The average number of children in the classrooms was 8 (S.D. = 2.4) and teachers rated all children’s social-emotional skills in fall and spring. Across the classrooms used in this study, enrollments varied based on agency’s grouping patterns and COVID-related low enrollments, but self-regulation scores were available for most children with ratings missing for only 1 or 2 children per classroom. The fall ratings were completed between October 2021 and November 2021, allowing teachers time to experience and know the children’s typical behaviors to produce valid ratings; the spring ratings were collected between April 2022 and May 2022.

Classroom observations were conducted between late November 2021 and March 2022; and did not overlap with the teachers’ ratings of children’s self-regulation. Data collectors attended a 4-day training in the fall of 2021 and completed a video certification to document reliability prior to data collection. To be certified as reliable, data collectors needed to agree on all items of the measure within 1 point on the 7-point scale (80%) and obtain at least 75% agreement in each of the three domains of the measure. Additionally, trained and reliable data collectors attended an in-person refresher training to review the observation guide prior to starting the classroom observations. Each classroom was observed one morning, with these observations lasting approximately 2–2.5 h. Following the procedure outlined by the developers of the classroom observation measure, observations consisted of six 10-min cycles with 5 to 10 min allocated between cycles for scoring and notetaking. Observers rotated observing and scoring each teacher present in the classroom and scored each cycle based on the observed teacher’s interactions with the enrolled infants and toddlers. After scoring the last cycle, observers rated “across the visit” items using evidence from the entire observation period.

### Measures

#### Self-regulation

To assess children’s social-emotional skills, teachers completed the Devereux Early Childhood Assessment for Toddlers (DECA-I/T; Mackrain et al., 2007) and preschoolers (DECA; LeBuffe and Naglieri, 1999). The DECA was designed by its developers to be a strengths-based approach to reliably measure within-child protective or resiliency factors (LeBuffe and Shapiro, 2004). The frequency of children’s behaviors is coded on a scale of 0 to 4 (Never to Very Frequently) for each item. Items fall under one of the following scales: initiative, attachment/relationship, and self-regulation. A combination of all items produces a Total Protective Factor score. For this study, the self-regulation scale was used which identifies children’s ability to appropriately regulate their emotions. The DECA-T form is designed for children between 18 and 36 months of age and contains 36 items while the DECA-PreK form is completed for children older than 36 months of age and was used in this study only for those children who were still enrolled in toddler classrooms. The DECA-T includes questions regarding a child’s self-regulation such as “Handle frustration well?,” “Calm herself/himself?,” and “Accept another choice when the first choice was not available?.” The DECA-PreK includes “Listen to or respect others?,” “Control his/her anger?,” and “Show patience?” as questions about a child’s self-regulation. The DECA-T self-regulation scale demonstrated excellent internal reliability (α = 0.92 for fall and 0.91 for spring) in this study. The internal reliability for the DECA PreK was also excellent (0.91 for fall and 0.95 for spring). Children’s standard scores for self-regulation were used in this study.

#### Classroom quality

To assess classroom quality, classroom observations were conducted using the Quality of Care for Infants and Toddlers (QCIT; Atkins-Burnett et al., 2015). This newer measure of classroom quality is a holistic classroom observational tool, specifically designed for infant-toddler care settings, that identifies evidence of classroom and caregiver practices for supporting children’s social-emotional, cognitive, and language and literacy development for children birth through 36 months of age. The Support for Social-Emotional Development domain encompasses items such as how the teacher responds to children’s social and emotional cues, supports social problem solving, and creates responsive routines, while Support for Cognitive Development documents the diversity of basic concepts taught and how teachers support children’s object exploration, scaffold problem solving, give choices, and extend pretend play. Support for Language and Literacy Development covers types of talk, features of talk, use of questions, and conversational turn-taking. During the observation, the observer asks the teacher to share *Good Night, Gorilla* with at least one child to capture a book share experience and attitudes toward books. As reported by the measure’s authors, the QCIT has good internal consistency for Supporting Social-Emotional Development (α = 0.87) and Supporting Cognitive Development (*α* = 0.84), and excellent internal consistency for Supporting Language and Literacy Development (*α* = 0.92) (Atkins-Burnett et al., 2015; Nguyen et al., 2022). Comparable to CLASS Toddler (La Paro et al., 2009), high-quality Early Head Start classrooms score similarly on both classroom measures (Xue et al., 2022). Following the procedures outlined by the developers of this measure (Atkins-Burnett et al., 2015; Nguyen et al., 2022), item-level scores were calculated for each item by averaging across cycles. To calculate domain scores, the average item scores and items scored “across the visit” were averaged for each domain.

#### Covariates

Child characteristics including gender (0 = female, 1 = male) and home language (0 = English, 1 = Spanish) were used as covariates in the final analytical models. Children’s race/ethnicity was not included due to collinearity with the home language variable. In addition, we did not include children’s age since we used DECA standard scores which reflect children’s age in the computed standard scores. Table 1 includes the descriptive statistics of variables used in this study.

### Analytical strategy

For this study, we used both classroom-level average self-regulation scores and individual-level self-regulation scores in the analytical models. We aggregated self-regulation skills of children who were enrolled in the same classroom in fall to examine how the classroom-level average of children’s self-regulation skills at the beginning of the year were associated with classroom quality. As noted previously, the number of children in a classroom ranged from 3 to 17 with an average of 8 children per classroom. The classroom-level average self-regulation scores in fall were used as a classroom-level predictor (i.e., level-2) for classroom quality. This study created a factor representing classroom quality using the three domains (Support for Social-Emotional, Cognitive, and Language Development) obtained from QCIT. We conducted a confirmatory factor analysis (CFA) to confirm that the classroom quality latent variables demonstrated acceptable loadings on the three domains.

**Figure 1:**
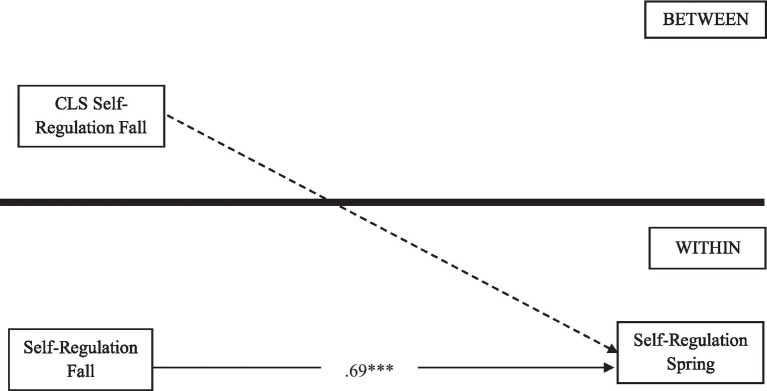
Multi-level direct model 1. Notes. ^***^p < 0.001; ^**^p < 0.01; ^*^p < 0.05. Solid lines indicate significant parameter estimates; standardized coefficients were provided. CLS, classroom; SE, social-emotional development; CD, cognitive development; LD, learning development. Control variables are included in this model; model fit: RMSEA =0.00, CFI =1.00.

To address the two research questions, we conducted multi-level models employing M*plus* (Muthén and Muthén, 1998–2017). For RQ1, we conducted a multi-level direct model by using the average of classroom-level self-regulation (level 2) as a predictor and gains in individual children’s self-regulation scores between fall and spring as an outcome. We included classroom ID in fall 2021 as a cluster ID and ran a two-level model with BETWEEN and WITHIN model commands. Individual children’s self-regulation skills in spring were regressed on the classroom-level average children’s self-regulation and children’s individual self-regulation skills in fall. We also controlled for children’s gender and home language in the direct model (see Figure 1).

**Figure 2:**
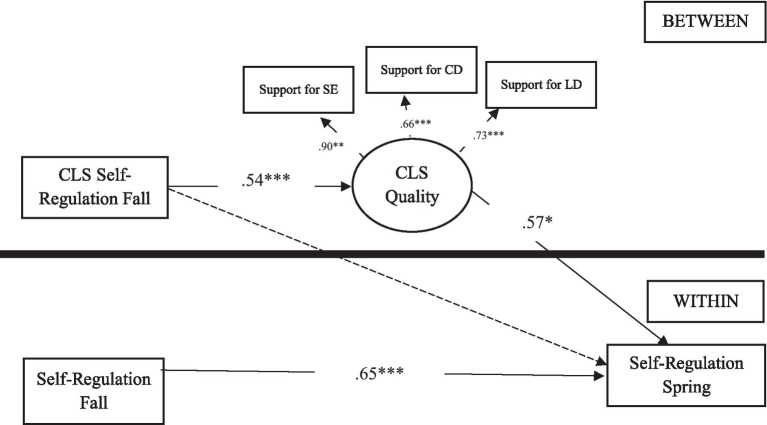
Multi-level mediation model. Notes. ^***^p < 0.001; ^**^p < 0.01; ^*^p < 0.05. Solid lines indicate significant parameter estimates; dashed lines indicate non-significant parameter estimates; Standardized coefficients were provided. CLS, classroom; SE, social-emotional development; CD, cognitive development; LD, learning development. Control variables are included in this model; model fit: RMSEA =0.05, CFI =0.95.

For RQ2, we examined how classroom quality (level-2) mediated between the initial classroom-level average children’s self-regulation (level-2) and individual children’s gain in self-regulation skills (level-1) across a year after controlling for children’s gender and home language. We used a multi-level mediation model to address this research question (see Figure 2). In the Mplus mediation model, level-2 variables were included in BETWEEN to examine a level-2 mediational path with an exogeneous variable (i.e., classroom-level self-regulation) and an endogenous variable (i.e., classroom quality) and the intercepts of level-1 outcome (i.e., self-regulation in spring). In WITHIN command, individual children’s self-regulation in fall, and gender and home languages were included as exogenous variables (i.e., predictors) and self-regulation in spring as an endogenous variable. We used the model constraint command to test the significance of the indirect paths from classroom-level children’s self-regulation skills to individual children’s gains in self-regulation skills through classroom quality (RQ2: 2–2-1 multilevel mediational path).

## Results

Table 1 provides descriptive statistics for the variables used in this study. We found that children’s self-regulation skills significantly increased between fall and spring (*t* = −2.48, *p* < 0.05). The classroom quality measure demonstrated acceptable factor loadings (0.66 – 0.90) to confirm the latent construct of the infant-toddler classroom quality measure. This model is a just-identified model, *χ*^2^ = 32.451, *df* = 3, *p* < 0.001, CFI = 1.00, TLI = 1.00, RMSEA =0.000.

The first model (see Figure 1) addressing RQ1 showed good model fit, CFI = 1.00, RMSEA = 00. Results show that classroom-level average self-regulation skills in the fall were not directly associated with the development of children’ self-regulation skills between fall and spring (β = − 0.01, *p* = 0.96).

The second model (see Figure 2) addressing RQ2 also showed acceptable model fit, CFI = 0.95, RMSEA = 0.05. Results indicated that classroom-level average self-regulation skills in the fall were positively associated with classroom quality (*β* = 0.54, *p* < 0.001), which, in turn, were positively associated with individual children’s self-regulation skill development between fall and spring (*β* = 0.57, *p* < 0.05). Children’s gender and home language were not associated with the development of self-regulation skills across a year.

## Discussion

Early care and education (ECE) classrooms have been recognized as an important context for young children’s learning and development (Shonkoff and Phillips, 2000; Bowman et al., 2001) that have demonstrated lasting impacts (NICHD Early Child Care Research Network, 2002, 2005; Vandell et al., 2010). Empirical evidence is accumulating that documents high-quality infant-toddler ECE can have significant short- and long-term effects on children’s development, especially for children who are growing up in low-income contexts (Campbell et al., 2001; Vandell et al., 2010; Yazejian et al., 2017; Meloy et al., 2019; Horm et al., 2022). Much of this research has focused on the nature and quality of ECE experience and associations with school readiness, early academic achievement, and behavior problems. Scant research has investigated the development of self-regulation, a critical component of resilience, with infants and toddlers in group care and education settings despite the increased attention self-regulation has received as a key mechanism predicting a variety of important outcomes (McClelland et al., 2015). With the relatively recent growth of enrollment of infants and toddlers in center-based ECE for significant amounts of time (Forry et al., 2018), in concert with the growing research base suggesting the key role self-regulation may play as a potent early life marker for later life success (Montroy et al., 2016), the purpose of this study was to fill a void in our understanding of the development of self-regulation in infant-toddler classrooms.

This study examined how the self-regulation skills of infants and toddlers in a classroom could potentially shape classroom interactions and quality which, in turn, could potentially shape the development of self-regulation skills of the individual infants and toddlers enrolled in a classroom across the ECE program year. Relative to our first research question, the results showed that classroom-level average self-regulation skills demonstrated by infants and toddlers in the fall did not directly predict individual children’s growth of self-regulation skills over a year. Our second research question investigated if classroom-level average children’s self-regulation skills related to classroom quality which, in turn, could promote gains in individual children’s self-regulation across the year. We found that this indirect path, rather than the direct path, best described the associations. Our results indicated that the average classroom-level self-regulation skills demonstrated by children in the fall were associated with levels of teachers’ support for social-emotional, cognitive, and language development in the winter which, in turn, associated with children’s increased growth in self-regulation skills from fall to spring. It is important to note that the classrooms included in this study were part of a statewide initiative to support and strengthen infant-toddler classrooms serving children growing up in the context of low-income and poverty, the children who research suggests most need support to develop their self-regulation skills (Wanless et al., 2011).

Our results for the first research question are contrary to past research findings obtained in studies of preschool-age children. For example, our findings with infants and toddlers differ from what Choi et al. (2018) found with preschool age children. Choi et al. (2018) found that preschoolers in classrooms with peers demonstrating higher classroom averages of self-regulation skills at the beginning of the year showed larger gains in teacher-reported self-regulation skills by the end of the school year. Montroy et al. (2016) found similar results in their study of the impact of peers on both self-regulation and literacy growth with preschoolers. One possible explanation for our differing findings is the developmental characteristics of infants-toddlers and preschoolers. It is possible that preschoolers are more attuned to peers, especially at the beginning of the year, than infants and toddlers. Another potential explanation is the consideration of classroom quality in the studies conducted by Choi et al. (2018) and Montroy et al. (2016). Choi et al. (2018) used classroom quality as a covariate. Montroy et al. (2016) did not include classroom quality as a variable in their study. As discussed in the introduction, past research and theory highlight the importance of individual characteristics, in this case the developmental differences of infants and toddlers versus preschoolers, in tandem with features of the context, in this case classroom quality, in supporting the development of self-regulation. As noted previously, the lack of studies focused on center-based classroom settings for infants and toddlers was one of the justifications for this study.

The results for the second research question supported our prediction that classroom quality would mediate the association between the classroom-level average of self-regulation skills demonstrated by infants and toddlers in the fall and the ongoing development of self-regulation skills in these individual children over the program year. This study illustrates how the infant-toddler classroom context can be potentially shaped by the enrolled children and the responses of a skilled teacher. Our findings suggest that teachers likely adjusted their levels of support for social-emotional, cognitive, and language development based on the average level of self-regulation demonstrated by children in their classroom in the fall. In classrooms where children had high self-regulation in the fall, the teacher could devote time to higher-level skills development and not focus on disruptions or issues associated with children’s low self-regulation. Thus, children’s higher average fall self-regulation levels likely contributed to producing a classroom environment that led to opportunities to build more skills. As noted by McClelland et al. (2015) when discussing findings with preschoolers, strong self-regulation skills position children to control their feelings, thoughts, and behaviors so they are able to take advantage of the instructional and learning opportunities available in their classrooms and school contexts. While not negating the importance of the family context, this study shows the importance of the classroom context, specifically a high-quality ECE classroom, on the development of self-regulation in infants and toddlers growing up in families with low incomes.

Relative to theory, Developmental Systems Theory (DST) and several other theories representing different disciplinary perspectives including Relational-Developmental-Systems (RDS; Overton and Lerner, 2012) from child psychology and the Bioecological Model of Human Development (Bronfenbrenner and Morris, 2006) used in the study of human development provide relevant frameworks to explain the development of self-regulation. Across these theoretical perspectives the development of self-regulation is posited to involve the dynamic interchange among characteristics of the person, in this case the individual infant or toddler; the context at multiple levels, in this case the classroom context shaped by the children and teachers; and relationships within and between those contexts over time, in this case over a year in an infant-toddler classroom. Researchers focused on risk tend to view self-regulation as a resilience factor adopting the DST perspective. As noted by Masten and Barnes (2018), an important implication of this perspective is that an individual’s resilience is not contained within the body and mind of that individual but depends on their connections to other people and systems external to the individual and mediated through relationships. Age-related changes in self-regulation as well as individual differences in self-regulation at a given age or developmental stage play fundamental roles in shaping children’s experiences and the responses that children bring forth from caregivers and others (Blair, 2009), which further shape self-regulation.

### Study limitations and strengths

Several limitations of the current study should be noted. First, given the use of a secondary dataset that was created for program evaluation purposes and not purposely designed to investigate the development of self-regulation in infant-toddler classrooms contexts, our study was limited to the available data. As noted previously, we had limited demographic information on the sample. Future research designed specifically to investigate the development of self-regulation in infant-toddler classroom contexts with diverse samples is needed to deepen our understanding.

Second, and relatedly, although measures of self-regulation are limited, especially those designed for use with infants and toddlers, our study would have been strengthened with additional measures of self-regulation. Although the DECA adopts a strengths-based approach consistent with our view of self-regulation as an important component of resilience and demonstrated strong internal validity in our study, it is based on teachers’ ratings of children’s behavior and thus shares the limitation of potential bias common to all behavior ratings. Although teacher ratings are often used to capture children’s social-emotional development, including self-regulation, this approach has known limitations. The teacher ratings used in this study could potentially be influenced by teachers’ perceptions, dispositions, or biases and thus may not be a pure measure of children’s self-regulation skills. Given that young children’s self-regulation skills can be observed and assessed, alternative methods such as direct observation could serve as effective measures for evaluating the self-regulation abilities of infants and toddlers in group care settings. Furthermore, incorporating parental reports of their children’s self-regulation skills could offer a comprehensive multi-informant assessment of self-regulation abilities in infants and toddlers. Future research incorporating these and other approaches to the measurement of self-regulation in infants and toddlers will likely broaden our understanding.

Third, our study could not isolate the influence of teachers versus peers on the development of self-regulation in the context of the studied classrooms. It is acknowledged that both teacher-child and child–child interactions contribute to classroom quality, the experiences of individual children in the classroom context, and the development of self-regulation in older preschoolers (Choi et al., 2018). Methods to disentangle these components of classroom experience is an area ripe for future research and development.

Fourth, the data used in this study were obtained from centers participating in a statewide initiative to enhance infant-toddler programming. Thus, it is possible that they may have offered different, potentially better, services than infant-toddler programs not participating in such an initiative. The average QCIT scores reported on Table 1 for the classrooms used in this study are similar to, but slightly higher than those reported by the QCIT’s developers (Atkins-Burnett et al., 2015). But, we need to acknowledge that the classrooms included in this study may offer programming that is not typical for infants and toddlers in the US, especially those enrolling children from low-resource contexts (Ruzek et al., 2014). Thus, generalizability of our findings could be limited and considered program specific because of the professional development and support the classrooms in our study were receiving.

Last, given this was an observational study that investigated associations, causality should not be implied. More research is needed to build the evidence base and deepen our understanding of the development of self-regulation in infants and toddlers, especially in varied contexts including classrooms. As an example, a bidirectional research design, encompassing both classroom quality and children’s self-regulation in both fall and spring, can offer a more nuanced understanding of the causal relationships between classroom quality and the development of children’s self-regulation.

Despite these limitations, our study had notable strengths that position it to make contributions to the existing body of literature. First, our study investigated the development of self-regulation, a skill that has received heightened attention as a key mechanism predicting a variety of important outcomes (McClelland et al., 2015), in an understudied age group, infants and toddlers. Beyond the focus on the development of an important skill in a unique age group, our study considered an understudied context, the early childhood classroom. Even with the body of the more prevalent preschool studies, it has been observed that the focus has generally not be placed on the ECE classroom context. In fact, Montroy et al. (2016) have called for more investigation of early school contexts to better understand the development of self-regulation. Our study focused on the context of infant-toddler center-based classrooms, using a relatively new tool that provided nuanced information about the nature and quality of the interactions and opportunities the children experienced. As discussed above, infant-toddler classrooms are an important context given the growth in infant-toddler group care and the amount of time increasing numbers of children spend in these settings (Forry et al., 2018).

### Implications for practice

Our results suggest that teachers adjusted their levels of support for social-emotional, cognitive, and language development based on the average level of self-regulation demonstrated by the infants and toddlers in their classrooms in the fall. Our inference is that the teachers whose classrooms scored high on the QCIT measure of quality were skilled in developmentally appropriate practices (NAEYC, 2020) which guide teachers to assess and incorporate information about children’s developmental skill levels and unique characteristics into their planning and implementation of classroom experiences and learning opportunities. Indeed, the lead teachers participating in this study were involved in a statewide initiative that provided training and other supports to improve and enhance infant-toddler classrooms. However, this is not typically the case. Past research has documented that teacher-child interactions and instructional support are typically lower quality in infant-toddler compared to preschool classrooms (La Paro et al., 2014; King et al., 2016). Infant-toddler teachers typically have lower levels of formal education than preschool, PreK, and K-12 teachers (Madill et al., 2016) with the infant-toddler workforce having some college but no degree (36%), or a high school diploma (28%) with fewer having an AA (17%) or bachelor’s degree or higher (19%) (NSECE, 2013). Additionally, fewer professional development (PD) opportunities are available that are tailored to the infant-toddler age group than those available for teachers who work with older children and research suggests the infant-toddler PD that is available is often delivered in formats that have questionable effectiveness and cover content focused on very basic skills (Madill et al., 2016). Pre-service preparation in institutions of higher education also has limitations with few infant-toddler focused courses, practicum or field experiences, or degree programs available (Horm et al., 2013; Chu, 2016). Thus, the findings of this study suggest what is possible with the supports of a state initiative offering ongoing and appropriate PD with content tailored to the infant-toddler age group receiving group care and education in center-based classrooms. Unfortunately, as documented above, this is not what is commonly available to infant-toddler teachers. Fewer PD opportunities are available for infant-toddler teachers overall and what is available is often offered as stand-along sessions without ongoing support (Madill et al., 2016).

A common observation is that the infant-toddler workforce is under-valued, under-compensated, under-professionalized, and under-supported (NSECE, 2013; Whitebook et al., 2016; Kwon et al., 2019; Vallotton et al., 2019). This exists despite the growing recognition that birth to age 3, as a unique developmental period, serves as the foundation for later development (Horm et al., 2016; Chazan-Cohen et al., 2017). The unique developmental characteristics of infants and toddlers, combined with the malleable nature of their development, necessitates that the adults in their lives, including nonparental caregivers such as infant-toddler teachers, are knowledgeable about how best to support and facilitate individual development. The findings of this study highlight the importance of infant-toddler teachers understanding how to implement developmentally appropriate classroom practices and ensure high-quality experiences tailored to the rapidly changing developmental skills of infants and toddlers. The measure of classroom quality used in this study captures how teachers adapt to the wide range of skills exhibited in infant-toddler classrooms and adjust to meet the needs of the enrolled infants and toddlers. Relative to self-regulation, strategies that are appropriate for non-mobile or pre-verbal infants may not be helpful or appropriate for a mobile infant and those with more language skills. Our results suggest that teachers who were skilled in creating a developmentally appropriate classroom supported the development of self-regulation in infants and toddlers through offering a higher quality of classroom experience. The higher quality classroom setting was associated with the further development of self-regulation skills in the enrolled infants and toddlers. Given the current nature of the infant-toddler workforce, this level of teacher expertise is a lofty expectation in most settings. Others have sounded the call for reform, but efforts to professionalize and appropriately compensate the infant-toddler workforce will take time. In the meantime, our findings suggest the development of classroom activities, curriculum, and associated PD to enhance quality in infant-toddler classrooms and expand teachers’ knowledge of child development, including the development of self-regulation, is paramount. Morawska et al. (2019) published an overview of the role of parenting in the development of self-regulation in children that highlights the evidence base for parenting interventions designed to promote the development of self-regulation in children under age 8, including a focus on infants and toddlers. The ECE field would profit from a similar review. Although some resources are available, a more intentional focus on accessible and easily implemented resources is crucial given the likely role of high-quality classrooms in supporting self-regulation development in conjunction with the current status of the infant-toddler workforce. Once again, it is worth noting that the classrooms in this study were drawn from centers that served infants and toddlers growing up in low-income contexts. In an analysis focused on a sample of toddlers from ECLS-B, Ruzek et al. (2014) found that toddlers in nonparental care typically experienced medium quality care (61%) rather than high quality (26%) or low quality (13%) care. Additionally, they found that children from low-resource homes that were enrolled in out-of-home care were more likely than their non-low-income counterparts to be in lower quality care. While the quality of care is important for all children and families (Vandell et al., 2010), it is especially important for young children growing up in the contexts of poverty and associated developmental risks because research has shown these children gain the most from high-quality care (La Paro et al., 2014). Thus, resources that are easy to access and use are critically important given the more typical low resource programs many infants and toddlers growing up in low-income contexts experience.

### Implications for research

The topic of self-regulation as a protective factor that promotes resilience as early as the infant and toddler years is important, including examination of its development within early care and education settings. Thus, the observation that little research has focused on the development of self-regulation in infant-toddler classrooms, to date, calls for more research. Additionally, the findings of the one existing published study we located (Salminen et al., 2021) that studied associations between teacher–child interaction quality, as measured by the CLASS-Toddler (La Paro et al., 2009), and several aspects of children’s self-regulation in Finnish and Portuguese toddler classrooms suggests a deeper and more nuanced examination is needed. Interestingly, the results found by Salminen et al. (2021) regarding which aspects of teacher behavior, engaged support for learning or emotional and behavioral support, associated with specific components of child self-regulation including attention and inhibitory control varied by country. While documenting that features of teachers’ classroom interactions can promote the development of children’s self-regulation skills overall, their findings indicate more research is needed in different sociocultural contexts and with measurement tools that offer more specificity in children’s developing skills and teachers’ classroom interactions.

Our study used a relatively new measure of infant-toddler classroom quality—the Quality of Care for Infants and Toddlers (QCIT; Atkins-Burnett et al., 2015). The availability of the QCIT, which was specifically developed to measure the quality of caregiver-child interactions for infants and toddlers across group settings, including center-based care and family child care homes, has implications for future research. The QCIT was developed based on an extensive review of the caregiver-child interaction literature and measures three important domains of caregiver support including social-emotional, cognitive, and language/literacy development, as well as areas of concern. The goal of the developers was to provide “early childhood professionals and researchers the means to obtain a better understanding of how caregivers and young children interact in child care settings and improve child care services in the future” (Atkins-Burnett et al., 2015, p. 1). As a tool developed specifically for infant-toddler ECE settings, it offers more nuanced information than other tools that were adapted for infant-toddler settings after being originally designed for classrooms serving older children. We advocate that the QCIT is a contemporary, comprehensive, and research-based tool that offers fresh insights on infant-toddler settings. For example, the existing data used in this study restricted our investigation to social-emotional aspects of development given the DECA was the only child outcome measure available to us. With its comprehensive focus including indicators of teachers’ support for cognitive and language development, and inclusion of areas of concerns across the observational visit, the QCIT offers possibilities for similar studies investigating other important child outcomes. Additionally, because the QCIT can be used in both center- and home-based programs, it expands research opportunities. Thus, the QCIT is positioned to contribute to and enrich the existing scant body of infant-toddler research examining impacts of early group ECE experiences, both center- and home-based.

Given the importance of self-regulation as a critical component of resilience, the field would profit from additional studies that deepen understanding of the mechanisms supporting its early development. We took advantage of an existing data set to explore associations of characteristics of enrolled infants and toddlers and features of their classroom context. Future research using different measures, designs, analytical strategies, and diverse samples and contexts is needed to further explain young children’s development of self-regulation. For example, different measures of infant-toddler self-regulation and classroom quality could be used. Longitudinal designs following the effects of various classroom characteristics on young children’s self-regulation development over time are needed. Analyses that test moderation as well as mediation and include diverse samples and classroom contexts are also needed. Relative to classroom context, it is important to investigate different grouping strategies such as mixed-age grouping that is often used in infant-toddler settings. Additional studies could investigate individual children’s experiences with teachers and control for parenting practices to further our understanding. These few examples illustrate the many open questions in the current literature.

## Conclusion

Resilience is a process that develops as a complex transaction as children experience their social-ecological contexts. This study examined one critical aspect of resilience, self-regulation, and how it potentially develops in infant-toddler classrooms. Most previous research investigating the development of self-regulation has been conducted with preschool age or older children and thus our focus on infants and toddlers is unique. The focus on infant-toddler classrooms is also a contribution given previous research emphasized the family context due to parents being acknowledged as the primary caregivers during children’s earliest years. This study expands the focus to consider other important caregivers and contexts by investigating how classroom quality, notably teacher-child interactions, may play a role in promoting resilience for infants and toddlers. The findings have implications for informing how to potentially support the development of self-regulation in more young children, especially those growing up at developmental risk, by improving the support and professional development of infant-toddler teachers which, in turn, should improve classroom quality and bolster the ability of infant-toddler programs to serve promotive (Masten and Barnes, 2018) functions in building resilience in infants and toddlers. To deepen understanding, additional research is needed to examine the development of not only self-regulation but other components of resilience in infants and toddlers in other important contexts including family, early care and education settings, neighborhoods, and other significant contexts.

## Data availability statement

The datasets used in this study are not publicly available due to human subjects and contractual restrictions. Requests to access the datasets should be directed to DH at dhorm@ou.edu.

## Ethics statement

The studies involving humans were approved by the University of Oklahoma Institutional Review Board. The studies were conducted in accordance with the local legislation and institutional requirements. Written informed consent for participation was not required from the participants or the participants’ legal guardians/next of kin in accordance with the national legislation and institutional requirements.

## Author contributions

DH: Funding acquisition, Project administration, Supervision, Writing – original draft, Writing – review & editing. SJ: Conceptualization, Data curation, Formal analysis, Methodology, Supervision, Writing – original draft, Writing – review & editing. DR: Data curation, Investigation, Writing – original draft, Writing – review & editing. SC: Conceptualization, Investigation, Supervision, Writing – review & editing.
